# Supplementary material for: Report of Hermansky–Pudlak Syndrome in Two Families with Novel Variants in HPS3 and HPS4 Genes
Source: Genes (Basel). 2023 Jan 5;14(1):145. doi: 10.3390/genes14010145 (PMC9858993; doi:10.3390/genes14010145)
Supplement: Supplementary file 1 [file genes-14-00145-s001.zip › genes-2104317-supplementary.pdf]

Supplementary Data

Table S1: All HPS with their classifications, respective phenotypes reported in literature and genes. + reported in disorder, - Not present in disorder, n/r not reported/ not observed in literature

S/N	Clinicals/ Phenotypes	HPS 1	HPS 2	HPS 3	HPS 4	HPS5	HPS 6	HPS7	HPS8	HPS9	HPS 10	HPS11
1	Ocular albinism	+	+	+	+	+	+	+	+	+	+	+
2	Hypopigmentation of retina	+	n/r	+	+	n/r	n/r	n/r	+	+	-	+
3	Hypopigmentation of fundus	+	+	n/r	n/r	n/r	+	n/r	+	+	-	n/r
4	Reduced visual acuity	+	+	+	+	+	+	+	+	-	-	+
5	Horizontal nystagmus	+	+	+	+	+	+	+	+	+	+	+
6	Photophobia	+	+	+	+	n/r	+	n/r	n/r	+	-	+
7	Strabismus	+	+	n/r	n/r	+	+	n/r	n/r	+	-	+
8	Iris transillumination	+/-	+	+	+	+	+	n/r	+	+	-	+
9	Marked choroidal reflex	n/r	-	n/r	n/r	n/r	+	n/r	n/r	n/r	n/r	n/r
10	Foveal hypoplasia	+	+	-	+	+	n/r	n/r	+	+	+	+
11	Skin pigment dilution, mild to severe, relative to unaffected family members	+	fair	+	+	+	+	n/r	+	+	+	+
12	Freckles on sun exposed areas	+	n/r	n/r	n/r	n/r	n/r	n/r	n/r	n/r	n/r	+
13	Hair pigment, mild to severe, dilution relative to unaffected family members	White	Fair	White	White	White	White	Golden	Blondie	Golden	White	Golden
14	Normal number of melanocytes	n/r	n/r	n/r	+	n/r	n/r	n/r	n/r	n/r	n/r	n/r
15	Reduced amount of melanin pigment in melanocytes	n/r	n/r	n/r	+	n/r	n/r	n/r	+	n/r	n/r	n/r

16	Weakly pigmented basal cell layer	n/r	n/r	n/r	+	n/r	n/r	n/r	n/r	n/r	n/r	n/r
17	Accumulation of ceroid pigment in perivascular macrophages	n/r	n/r	n/r	+	n/r	n/r	n/r	n/r	n/r	n/r	+
18	Bleeding diathesis	+	+	+	+	+	+	+	+	+	+	+
19	Easy bruising	+	+	+	+	+	+		+	+	+	+
20	Epistaxis	+	+	-	+	+	+	-	+	+	+	+
21	Gingival bleeding	+	+	n/r	n/r	n/r	n/r	n/r	+	+	+	+
22	Menorrhagia	n/r	+	+	+	+	+	n/r	+	n/r	+	+
23	Normal platelets count	+	n/r	n/r	-	-/+	-	n/r	n/r	-	n/r	n/r
24	Absence of platelet dense bodies	+	+	+	+	+	+	+	+	+	n/r	+
25	Lack of secondary aggregation response of platelets	n/r	n/r	+	+	n/r	n/r	+	+	+	n/r	+
26	Pulmonary fibrosis	+	+	-	+/++	-	-	-	-	-	+	-
27	Restrictive lung disease	+	-	-	+	-	-	-	-	-	+	-
28	Frequent upper respiratory tract infection	+	+	-	+	-	+	-	-	-	+	-
29	Granulomatous colitis	+	-	-	+	-	-	+	+	n/r	-	+
30	Abdominal pain	+	-	-	+	n/r	n/r	n/r	+	n/r	-	+
31	Inflammatory bowel disease	+	-	-	+		-	n/r	n/r	n/r	-	n/r
32	Bloody diarrhea	+	-	-	+	n/r	n/r	n/r	+	n/r	-	n/r
33	Neutropenia	-	+	-	-	-	-	n/r	-	+	+	-
34	Decreased numbers of NK cells	-	+	-	-	-	-	n/r	n/r	+	+	-
35	Recurrent bacterial infection	+	+	-	-	-	-	n/r	-	+	+	-

39	Broad nasal root	-	+	-	-	-	n/r	n/r	-	-	-	-
40	Thin upper lip	-	+	-	-	-	-	n/r	-	-	-	-
41	Cardiomyopathy	+	-	-	-	-	-	n/r	-	n/r	-	n/r
42	Microcephaly	-	+	-	-	-	-	n/r	-	-	+	-
43	Mental retardation	-	+	-	-	-	-	-	-	-	-	-
44	Reported Causative Gene	*HPS1/BLOC3S1*	*AP3B1/ADTB2*	*HPS3/BLOC2S1*	*HPS4/BLOC3S2*	*HPS5/BLOC2S2*	*HPS6/BLOC2S3*	*HPS7/DTNBP1/ BLOC1S8*	*HPS8/BLOC1S3/ BLOS3*	*HPS9/BLOC1S/ PLDN/ BLOS6*	*HPS10/AP3D1/ ADTD*	*HPS11/BLOC1S5*
45	Human locus	*10q24.2*	*5q14.1*	*3q24*	*22q12.1*	*11p15.1*	*10q24.32*	*6p22.3*	*19q13.32*	*15q21.1*	*19p13.3*	*6p24.3*
46	Protein Name	HPS1	AP3B1	HPS3	HPS4	HPS5	HPS6 Protein	Dysbindin/ HPS7	BLOC1S3/ HPS8	BLOC1S6/ Pallidin/HPS[illegible]	AP3D1	BLOCS5
47	UniProtKB ID	Q92902	O00203	Q969F9	Q9NQG7	Q9UPZ3	Q86YV9	Q96EV8	Q6QNY0	Q9UL45	O14617	Q8TDH9

Table S2A: HPS3 biogenesis of lysosomal organelles complex 2 subunit 1 (NM_0323383.5), chromosomal location 3q24, also known as BLOC2S1, SUTAL. A total 44 mutations are reported in literature that cause Hermansky Pudlak Syndrome. The 44 mutations include 16 missenses, 10 splice substitution, 10 small deletion, 3 small deletion/duplications, 2 in deletions, 2 gross deletion, and 1 gross insertion mutations.

Missense/nonsense 16 mutations

S/No	Nucleotide	amino acid change	Reference
1	c.7C>T	Gln3Term	Lecchi (2019) Platelets , 1
2	c.15C>G	Tyr5Term	Huizing (2020) Hum Mutat 41, 543
3	c.319C>T	Arg107Term	Okamura (2019) Pigment Cell Melanoma Res 32, 848
4	c.479G>A	Ser160Asn	Almazni (2020) Hum Mutat 41, 1848
5	c.691C>T	Arg231Term	Liu (2021) Pigment Cell Melanoma Res 34, 111

6	c.796G>A	Glu266Lys	Fromer (2014) Nature 506, 179
7	c.868C>T	Gln290Term	Khan (2016) Clin Genet 90, 96
8	c.1012G>T	Glu338Term	Huizing (2020) Hum Mutat 41, 543
9	c.1189C>T	Arg397Trp	Huizing (2001) Am J Hum Genet 69, 1022<br>Capalbo (2019) PLoS Genet 15: e1008409<br>Okamura (2019) Pigment Cell Melanoma Res 32: 848
10	c.1509G>A	Met503Ile	Yousaf (2016) Pigment Cell Melanoma Res 29, 231
11	c.1673T>C	Leu558Pro	Lasseaux (2018) Pigment Cell Melanoma Res ,
12	c.1838C>G	Ser613Term	Wei (2019) Pigment Cell Melanoma Res 32, 373<br>Liu (2021) Pigment Cell Melanoma Res 34: 111
13	c.1870G>T	Glu624Term	Huizing (2020) Hum Mutat 41, 543<br>Turro (2020) Nature 583: 96
14	c.2464C>T	Arg822Term	Bastida (2019) Ann Med epub, epub<br>Bastida (2018) Haematologica 103: 148
15	c.2804G>A	Trp935Term	Liu (2021) Pigment Cell Melanoma Res 34, 111
16	c.2805G>A	Trp935Term	Wei (2016) Pigment Cell Melanoma Res 29, 702<br>Liu (2021) Pigment Cell Melanoma Res 34: 111

Splicing mutation

S/No	Nucleotide	Splicing mutation	Reference
17	c.712+2T>C	IVS2 ds T-C +2	Wei (2016) Pigment Cell Melanoma Res 29, 702<br>Liu (2021) Pigment Cell Melanoma Res 34: 111
18	c.885-1G>A	IVS3 as G-A -1	Thielen (2010) J Thromb Haemost 8, 1643
19	c.1163+1G>A	IVS5 ds G-A +1	Huizing (2001) Am J Hum Genet 69, 1022<br>Xiong (2015) Science 347: 1254806
20	c.1691+1G>A	IVS9 ds G-A +1	Huizing (2020) Hum Mutat 41, 543
21	c.1691+2T>G	IVS9 ds T-G +2	Huizing (2001) Am J Hum Genet 69, 1022
22	c.2482-2A>G	IVS13 as A-G -2	Huizing (2001) Am J Hum Genet 69, 1022<br>Xiong (2015) Science 347: 1254806
23	c.2589+1G>C	IVS14 ds G-C +1	Huizing (2001) Am J Hum Genet 69, 1022<br>Xiong (2015) Science 347: 1254806
24	c.2589+1G>T	IVS14 ds G-T +1	Huizing (2020) Hum Mutat 41, 543
25	c.2796+3A>G	IVS15 ds A-G +3	Liu (2021) Pigment Cell Melanoma Res 34, 111
26	c.2888-1612G>A	IVS16 as G-A -1612	Huizing (2001) Am J Hum Genet 69, 1022<br>Vorechovsky (2010) Hum Genet 127: 135

Small deletions: 10 mutations

S/No	Nucleotide	Deletion mutation	Reference

27	c.437_439delGAG	p.(Gly146del)	Okamura (2019) Pigment Cell Melanoma Res 32, 848
28	c.851_852delGA	p.(Arg284Lysfs*11)	Huizing (2020) Hum Mutat 41, 543
29	c.1153_1160delGTCATTAC	p.(Val385Lysfs*2)	Trujillano (2017) Eur J Hum Genet 25, 176
30	c.1291delC	p.(Leu431Phefs*3)	Okamura (2019) Pigment Cell Melanoma Res 32, 848
31	c.1631delA	p.(Lys544Argfs*52)	Liu (2021) Pigment Cell Melanoma Res 34, 111
32	c.2208_2209delTC	p.(Gln737Alafs*20)	Wei (2016) Pigment Cell Melanoma Res 29, 702 Liu (2021) Pigment Cell Melanoma Res 34: 111
33	c.2628delT	p.(Ile877Phefs*25)	Lasseaux (2018) Pigment Cell Melanoma Res ,
34	c.2733delG	p.(Leu912*)	Huizing (2020) Hum Mutat 41, 543
35	c.2739_2742delGAGA	p.(Glu913Aspfs*14)	Huizing (2020) Hum Mutat 41, 543
36	c.2771delA	p.(Asn924Ilefs*4)	Sandrock-Lang (2017) Blood Cells Mol Dis 67, 75

Small insertions: 3 mutations

S/No	Nucleotide	Protein	Reference
37	c.87dupG	p.(Arg30Alafs*2)	Saito (2020) J Dermatol 47, e18
38	c.726_727ins11	p.(Ile243Cysfs*41)	Huizing (2020) Hum Mutat 41, 543
39	c.1426dupA	p.(Ile476Asnfs*8)	Saito (2020) J Dermatol 47, e18

Small indels: 2 mutations

S/No	Nucleotide	Protein	Reference
40	c.1107_1119del13insC	p.(Pro370_Ser373del)	Huizing (2020) Hum Mutat 41, 543
41	c.1195_1196delAGinsGGTGCAT	p.(Ser399Glyfs*18)	Huizing (2020) Hum Mutat 41, 543

Gross deletions: 2 mutations

S/No	DNA level	Description	Nucleotide	Protein	Reference
42	gDNA	3904 bp incl. ex. 1	Not yet available	Not yet available	Anikster (2001) Nat Genet 28, 376 Torres-Serrant (2010) J Pediatr Hematol Oncol 32: 448
43	cDNA	c.89_114	c.89_114del26	p.(Arg30Leufs*42)	Liu (2021) Pigment Cell Melanoma Res 34, 111

Gross insertions: 1 mutation

S/No	DNA level	Insertion/ duplication	Description	Reference

44	gDNA	Duplication	41 bp, c.1555_1595	Power (2019) Orphanet J Rare Dis 14, 52

Table S2B: HPS4 biogenesis of lysosomal organelles complex 3 subunit 2 (NM_022081.6), chromosomal location 22cen-q12.3, also known as BLOC3S2 and LE. A total 39 mutations are reported in literature that cause Hermansky Pudlak Syndrome. The 39 mutations consist of 23 missenses, 5 splice substitutions, 5 small deletions, 5 small insertions / duplications, and 1 gross insertion.

Missense/nonsense: 23 mutations

S/NO	Nucleotide	HGMD amino acid change	Reference
1	c.45G>A	Trp15Term	Carmona-Rivera (2011) J Invest Dermatol 131, 2394<br>Okamura (2018) Pigment Cell Melanoma Res 31: 267
2	c.123T>A	Tyr41Term	Okamura (2018) Pigment Cell Melanoma Res 31, 267
3	c.148C>T	Gln50Term	Wei (2019) Pigment Cell Melanoma Res 32, 373<br>Liu (2021) Pigment Cell Melanoma Res 34: 111
4	c.272T>C	Leu91Pro	Bastida (2019) Ann Med epub, epub<br>Bastida (2018) Haematologica 103: 148
5	c.357C>G	Tyr119Term	Huizing (2020) Hum Mutat 41, 543
6	c.412G>T	Glu138Term	Anderson (2003) Hum Genet 113, 10<br>Xiong (2015) Science 347: 1254806
7	c.416G>A	Trp139Term	Power (2019) Orphanet J Rare Dis 14, 52
8	c.430G>T	Glu144Term	Arcot Sadagopan (2017) Ophthalmic Genet 38, 194
9	c.461A>G	His154Arg	Anderson (2003) Hum Genet 113, 10
10	c.541C>T	Gln181Term	Suzuki (2002) Nat Genet 30, 321<br>Saito (2013) Psychiatr Genet 23: 163<br>Xiong (2015) Science 347: 1254806
11	c.554G>A	Arg185His	Arcot Sadagopan (2017) Ophthalmic Genet 38, 194
12	c.649C>T	Arg217Term	Anderson (2003) Hum Genet 113, 10<br>Xiong (2015) Science 347: 1254806<br>Lozynska (2018) Exp Oncol 40: 73
13	c.664G>T	Glu222Term	Anderson (2003) Hum Genet 113, 10<br>Xiong (2015) Science 347: 1254806
14	c.667C>T	Gln223Term	Hou (2020) Proc Natl Acad Sci U S A 117, 3053
15	c.730C>T	Gln244Term	Araki (2014) J Dermatol 41, 186
16	c.803G>A	Arg268Lys	Lasseaux (2018) Pigment Cell Melanoma Res ,
17	c.1132C>T	Gln378Term	Huizing (2020) Hum Mutat 41, 543
18	c.1318C>T	Gln440Term	Sandrock-Lang (2018) Blood Cells Mol Dis 69, 113
19	c.1396C>T	Arg466Cys	Stearman (2019) Am J Respir Crit Care Med epub, epub

20	c.1546C>T	Gln516Term	Huizing (2020) Hum Mutat 41, 543
21	c.1856C>T	Pro619Leu	Lasseaux (2018) Pigment Cell Melanoma Res ,
22	c.1858C>T	Gln620Term	Sakata (2013) Respir Med Case Rep 9, 38
23	c.1891C>T	Gln631Term	Suzuki (2002) Nat Genet 30, 321<br>Xiong (2015) Science 347: 1254806

Splicing: 5 mutations

S/NO	Nucleotide	Splicing mutation	Reference
24	c.276+5G>A	IVS4 ds G-A +5	Yousaf (2016) Pigment Cell Melanoma Res 29, 231
O25	c.597-2A>T	IVS7 as A-T -2	Jones (2012) J Thromb Haemost 10, 306
26	c.596+1G>A	IVS7 ds G-A +1	Okamura (2019) Pigment Cell Melanoma Res 32, 848
27	c.706+1G>A	IVS9 ds G-A +1	Huizing (2020) Hum Mutat 41, 543
28	c.1713+5G>C	IVS11 ds G-C +5	Wei (2019) Pigment Cell Melanoma Res 32, 373<br>Liu (2021) Pigment Cell Melanoma Res 34: 111

Small deletions: 5 mutations

S/NO	Nucleotide	Protein	Reference
29	c.47delA	p.(Asn16Ilefs*11)	Carmona-Rivera (2011) J Invest Dermatol 131, 2394
30	c.57delT	p.(Leu20Phefs*7)	Suzuki (2002) Nat Genet 30, 321
31	c.61delT	p.(Tyr21Metfs*6)	Hou (2020) Proc Natl Acad Sci U S A 117, 3053
32	c.1547_1548delAG	p.(Gln516Argfs*42)	Arcot Sadagopan (2017) Ophthalmic Genet 38, 194
33	c.2054delC	p.(Pro685Leufs*17)	Bachli (2004) Am J Med Genet A 127A, 201<br>Bastida (2018) Haematologica 103: 148<br>Bastida (2019) Ann Med : 1

Small insertions: 5 mutations

S/NO	Nucleotide	Protein	Reference
34	c.630dupC	p.(Ala211Argfs*47)	Wu (2019) Medicine (Baltimore) 98, e16899
35	c.1102dupG	p.(Asp368Glyfs*4)	Deng (2018) Hum Mutat 39, 1238
36	c.1896_1897dupCG	p.(Val633Alafs*4)	Okamura (2019) Pigment Cell Melanoma Res 32, 848
37	c.1966_1967dupAC	p.(Ala657Argfs*46)	Stearman (2019) Am J Respir Crit Care Med epub, epub
38	c.2089_2093dupAAGCA	p.(Lys699Serfs*5)	Suzuki (2002) Nat Genet 30, 321

Gross insertions: 1 mutation

S/N0	DNA level	Insertion/ duplication	Description	Reference
39	cDNA	Duplication	24 bp nt. 949-972	Suzuki (2002) Nat Genet 30, 321
